# Cytokines in gingivitis and periodontitis: from pathogenesis to therapeutic targets

**DOI:** 10.3389/fimmu.2024.1435054

**Published:** 2024-08-26

**Authors:** Nicole Neurath, Marco Kesting

**Affiliations:** ^1^ Department of Oral and Cranio-Maxillofacial Surgery, Uniklinikum Erlangen, Friedrich-Alexander University Erlangen-Nürnberg, Erlangen, Germany; ^2^ Deutsches Zentrum Immuntherapie DZI, Uniklinikum Erlangen, Erlangen, Germany

**Keywords:** oral mucosa, periodontitis, cytokines, pathogenesis, cytokine targeting

## Abstract

Chronic inflammatory processes in the oral mucosa and periodontitis are common disorders caused by microflora and microbial biofilms. These factors activate both the innate and adaptive immune systems, leading to the production of pro-inflammatory cytokines. Cytokines are known to play a crucial role in the pathogenesis of gingivitis and periodontitis and have been proposed as biomarkers for diagnosis and follow-up of these diseases. They can activate immune and stromal cells, leading to local inflammation and tissue damage. This damage can include destruction of the periodontal ligaments, gingiva, and alveolar bone. Studies have reported increased local levels of pro-inflammatory cytokines, such as interleukin-1beta (IL-1beta), tumor necrosis factor (TNF), IL-6, IL-17, and IL-23, in patients with periodontitis. In experimental models of periodontitis, TNF and the IL-23/IL-17 axis play a pivotal role in disease pathogenesis. Inactivation of these pro-inflammatory pathways through neutralizing antibodies, genetic engineering or IL-10 function has been demonstrated to reduce disease activity. This review discusses the role of cytokines in gingivitis and periodontitis, with particular emphasis on their role in mediating inflammation and tissue destruction. It also explores new therapeutic interventions that offer potential for research and clinical therapy in these chronic inflammatory diseases.

## Background

**Figure 1:**
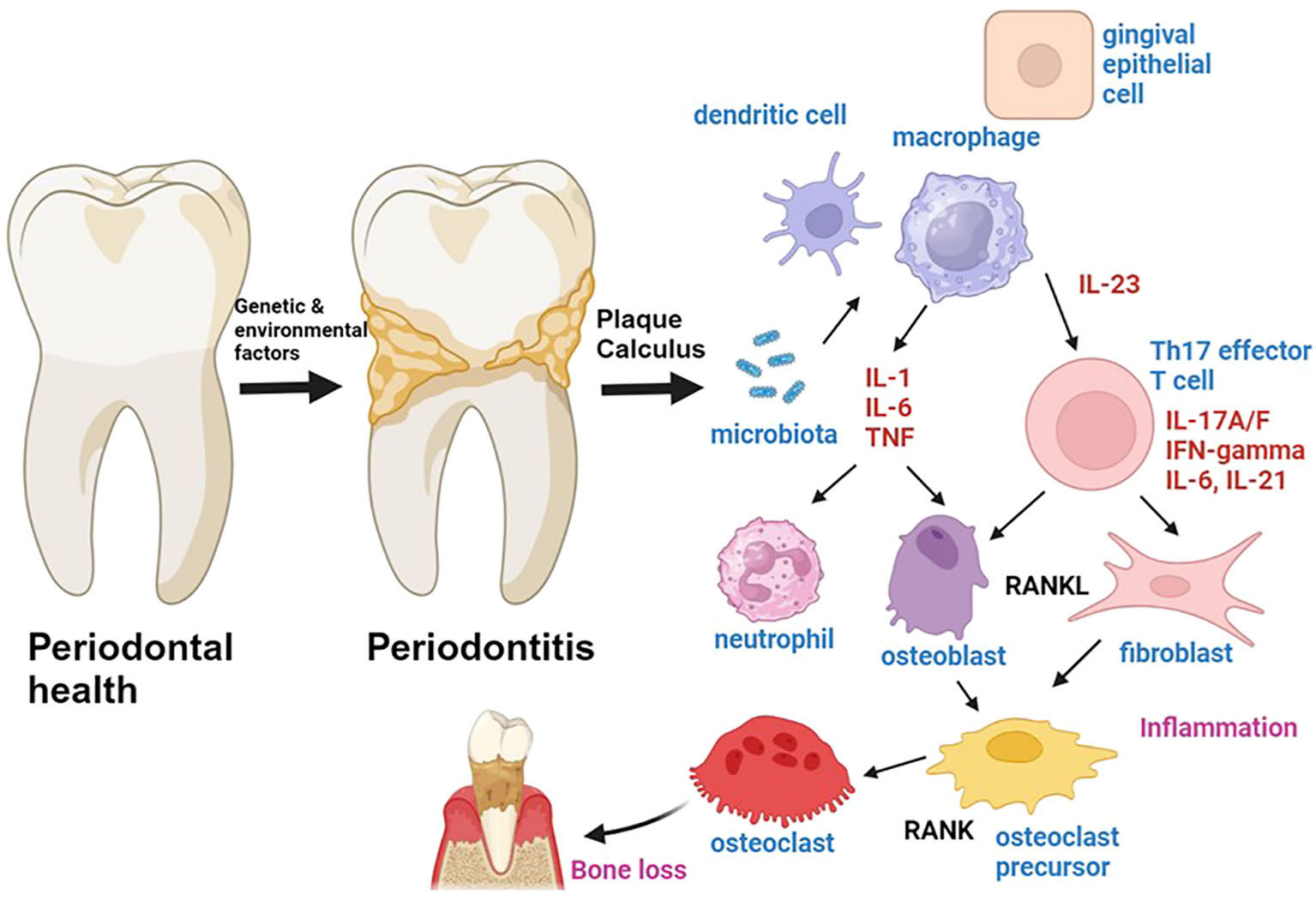
The role of cytokines in the pathogenesis of periodontitis is a topic of significant interest in the field of periodontal research. Genetic and environmental factors have been identified as predisposing factors for the development of periodontitis, which is a chronic inflammatory process involving the activation of both innate and adaptive immune cells. The microbiota-driven activation of innate immune cells leads to the production of pro-inflammatory cytokines such as IL-1, IL-6, TNF, and IL-23. IL-1, IL-6 and TNF are produced by antigen presenting cells such as macrophages, and promote inflammation by inducing chemotaxis and activation of neutrophils and by activating T cells and fibroblasts. IL-23 is produced by antigen-presenting cells and gingival epithelial cells. IL-1 and IL-23 induce the activation of Th17 cells, which can produce IL-17A/F, IFN-gamma, IL-21 and IL-6. Th17 cells induce the activation of RANKL expression by osteoblasts and fibroblasts, thereby inducing osteoclastogenesis and bone loss in periodontitis. Figure was created with BioRender.

The World Health Organization (WHO) has estimated that severe periodontal diseases affect approximately 19% of the global adult population, representing more than one billion cases worldwide (1). Periodontitis is primarily characterized by local inflammation of the periodontium and destruction of the alveolar bone that results from a pro-inflammatory host immune response to bacteria (**Figure 1**) (2–7). A recent French study revealed that only 19% of individuals at risk of severe periodontitis perceived themselves to be afflicted with gum disease (8). This indicates that periodontitis may frequently be misdiagnosed due to the low level of awareness among those at risk.

Dental plaque is an oral biofilm of microorganisms that continuously grows on the surfaces of teeth. This plaque plays an important role in both oral health and the development of diseases such as gingivitis and periodontitis (9). In 1994, Marsh proposed the “Ecological Plaque Hypothesis,” which postulates that periodontitis results from an imbalance in the total microflora due to ecological stress, leading to an enrichment of disease-related microorganisms (10). However, subsequent research has demonstrated that periodontitis is characterized by an imbalance between the types of microorganisms present in a person’s natural microflora in the oral cavity. This dysbiosis has been identified as a crucial factor in driving local inflammation in periodontitis. In 2020, a new theory on the etiology of periodontitis was formulated, which was designated the “Inflammation-Mediated-Polymicrobial-Emergence and Dysbiotic-Exacerbation” (IMPEDE) model. In this context, it was proposed that inflammation represents the consequence of the dysbiotic events that occur in the disease, which drive the transition from oral health to periodontitis (11).

Previous classifications have suggested that periodontitis can manifest in two distinct forms: chronic and aggressive periodontitis (2, 12). In this context, aggressive periodontitis was defined as a rapidly progressing form of periodontitis that is characterized by pronounced tissue destruction and possible edentulism in early life. In contrast, chronic periodontitis was defined as a usually more slowly progressing disease associated with relatively intense gingival inflammation and thick deposits of polymicrobial communities on affected root surfaces (13, 14). In 2017, a new classification scheme for periodontitis was introduced (15). The previous classification, which distinguished between “chronic” and “aggressive” forms, has been replaced by a classification in which all forms of periodontitis are now grouped under a single category, “periodontitis,” and are further characterized based on a multi-dimensional staging and grading system. This classification of periodontitis comprises four distinct stages (stage I-IV), which are defined by disease severity and complexity. The severity of the disease is stratified according to the level of interdental clinical attachment loss (at the site of greatest loss), radiographic bone loss, and tooth loss, while local complexity is assessed by measuring the maximum probing depth and bone loss. Furthermore, the extent and distribution of the disease (localized, generalized, or molar/incisor pattern) are included in this classification of periodontitis.

The periodontium is the collagenous structure that surrounds and attaches the teeth to the underlying alveolar bone, allowing for adequate esthetics and tooth function (16–18). There is a stepwise transition from non-inflamed gum and healthy periodontal tissue to progressive periodontitis (4, 19, 20). In the initial stages of periodontal disease, the soft tissues of the gum are affected. However, advanced periodontal disease not only affects the gums but also the bone structures supporting the teeth, leading to local inflammation that can affect the surrounding bone (14, 19, 20). Finally, periodontitis can progress to an advanced or severe stage, which is characterized by local abscesses, marked inflammation, and progressive bone loss.

The severity and complexity of the disease are classified based on objective parameters such as radiographic bone loss (RBL), tooth loss, maximal probing and pocket depth, interdental attachment level and clinical attachment loss (CAL) (21). Furthermore, the grading of periodontitis can predict the disease’s progression, responsiveness to standard therapy, and potential impact on systemic health (22). Grade A periodontitis is characterized by a lack of CAL and RBL and is associated with a slow rate of progression. On the other hand, grade B periodontitis is defined by RBL or CAL <2 mm over 5 years and biofilm deposits and has a moderate rate of progression. Finally, grade C periodontitis is characterized by marked evidence of disease progression, with RBL or CAL greater than or equal to 2 mm over 5 years, and significant biofilm deposits leading to tooth loss and bone resorption. Smoking, insufficient oral hygiene and diabetes are important disease modifiers that favor an a progressive course of periodontitis (23–25).

## Cytokines in general

Cytokines are small regulatory proteins with a wide variety of molecular weights, ranging from approximately 6 to 70 kDa (26). They are produced by both immune and non-immune cells and play a pivotal role in controlling tissue homeostasis and the activation of the body’s immune system (27–29). Cytokines act as key modulators within localized compartments and the systemic circulation in the body, regulating numerous crucial processes, including intercellular communication, hematopoietic development, and immune responses. They are important determinants of health (29–31). Moreover, they regulate dynamic host responses to infectious agents, tumor cells, tissue injury, and inflammatory factors (32–35). Cytokines are pleiotropic, meaning they can act on multiple targets and elicit multiple physiological effects simultaneously or sequentially. Consequently, a single cytokine can act on several cell types, including immune and non-immune cells, and induce a range of biological activities.

A multitude of diseases are associated with alterations in cytokine production, which may result in significant modulation of disease activity (36, 37). For example, the increased production of the cytokine TNF plays a fundamental role in numerous chronic inflammatory disorders such as psoriasis and rheumatoid arthritis. Suppression of TNF bioactivity by neutralizing antibodies has shown clinical efficacy in the treatment of these diseases (38, 39). Moreover, the overproduction of numerous cytokines has been implicated in organ failure and death. One illustrative example is the so-called cytokine storm, which has been demonstrated to be associated with a poor prognosis in critical cases of coronavirus disease 2019 (COVID-19) (40–43). Consequently, the levels of cytokines are recognized as potential modulators of immune cell activity. This finding has clinical relevance, as modulation of cytokine bioactivities is currently used in many chronic inflammatory diseases, including asthma, rheumatoid arthritis, inflammatory bowel disorders, and psoriasis, for clinical therapy (36, 44).

## Cytokines, MMPs and the OPG/RANKL system in periodontitis

In the healthy oral mucosa, a balance between cytokines with pro- and anti-inflammatory properties is important for tissue homeostasis (45). In gingivitis and periodontitis, elevated levels of pro-inflammatory cytokines have been identified in affected tissue, which shifts the balance between pro- and anti-inflammatory cytokines toward local inflammation (5, 46–48). Cytokine levels can even exceed those observed in other chronic inflammatory diseases, such as inflammatory bowel diseases. This is further supported by the observation that in patients with periodontitis and inflammatory bowel diseases, the expression of IL-17A/F and IFN-gamma was significantly increased in gingival tissue in comparison with intestinal mucosa. This suggests the presence of high cytokine levels in inflamed periodontal tissue that favor local inflammation and fibrosis (49).

Cytokines can also control bone resorption in the periodontal area. Specifically, they modulate the production of “receptor activator of NF-kappaB ligand” (RANKL), which is produced by osteoblasts, fibroblasts and lymphocytes and activates osteoclasts via RANKL/RANK interactions to induce bone resorption (**Figure 1**) (50, 51). This process is inhibited by osteoprotegerin (OPG), which binds to RANKL and prevents RANKL/RANK interactions (52). Thus, cytokines control pleiotropic key pathways of critical importance for health, bone and tissue homeostasis in the periodontal environment.

Periodontitis is characterized by an imbalance between pro- and anti-inflammatory cytokines due to microbial and host-derived factors, such as gram-negative bacteria, genetic factors and drugs (53–56). This leads to pro-inflammatory processes that promote local inflammation and alveolar bone loss (48). Microorganisms can invade and damage periodontal tissues by producing chemicals such as hydrogen sulphide and ammonia (16). They can also activate immune cells such as macrophages, dendritic cells and lymphocytes, as well as non-immune cells such as fibroblasts, epithelial cells and osteoclasts (5, 57). This activation process is triggered by bacterial products via Toll-like receptors on target cells or by bacterial antigens (58). It results in cell activation and the production of soluble mediators, such as cytokines and chemokines. Cytokines affect the inflammatory and destructive disease process in periodontitis through several pathways. Firstly, they activate cells of the innate and adaptive immune systems, causing them to proliferate and produce more pro-inflammatory cytokines (27, 28). Secondly, they activate RANKL expression and receptor binding, while suppressing OPG, leading to bone loss (59, 60). Finally, they induce the production of matrix-metalloproteinases (MMPs) by tissue resident cells such as fibroblasts (61–65). Matrix metalloproteinases (MMPs), including collagenases, matrilysins, and stromelysins, can degrade various proteins in the extracellular matrix, resulting in tissue destruction. In addition, chemokines as soluble mediators play an important role in the pathogenesis of chronic periodontitis. These regulatory proteins control the chemotaxis and migration of immune cells such as macrophages and lymphocytes, thereby amplifying the local inflammatory process in periodontitis. Taken together, these data suggest that cytokines play a key role in the pathogenesis of periodontitis by controlling the inflammatory pathways that drive local tissue inflammation and bone destruction.

## Systemic inflammation and general diseases in periodontitis

Several lines of evidence indicate that periodontitis may increase systemic levels of pro-inflammatory cytokines and may thereby influence inflammatory, degenerative or neoplastic processes. In experimental periodontitis, the systemic levels of IL-1beta and TNF were upregulated upon periodontal infection indicating periodontitis-induced systemic inflammation. This process augmented brain inflammatory responses and subsequently exacerbated Alzheimer’s disease-like pathology and cognitive decline in a murine model of Alzheimer´s disease (3 × Tg-AD mice) suggesting that periodontitis-driven inflammatory processes can augment neurodegenerative disorders (66). Moreover, cytokines derived from periodontal inflammation can deteriorate experimental arthritis. Specifically, the experimental periodontitis induced by infection with Porphyromonas gingivalis affected the experimental arthritis triggered by injection of bovine serum albumin (67). While arthritis had no influence on the alveolar bone resorption in periodontitis, arthritic mice exposed to Porphyromonas gingivalis exhibited higher TNF and IL-17 levels and demonstrated more joint damage than control mice. This effect was abrogated by IL-17RA deficiency, indicating that periodontitis induces cytokine-mediated signaling events that aggravate arthritis activity (67). An additional mechanism by which periodontitis controls inflammation in other organs is related to the function of IL-1 (68). Indeed, it has been demonstrated that experimental periodontitis induces maladaptive trained myelopoiesis, which is induced by elevated IL-1 levels. This aberrant myelopoiesis, in turn, predisposes for increased activity of experimental arthritis, suggesting that the inflammatory environment in periodontitis may contribute to systemic effects and significant comorbidities at other sites in the body (68). This concept leads to the hypothesis that treatment of periodontitis may affect the severity of other inflammatory diseases. A recent interventional study demonstrated that non-surgical periodontal therapy exhibited an additional effect over conventional dermatological treatment in reducing both the severity and the extent of psoriasis (69).

Another study demonstrated that periodontitis leads to increased serum levels of IL-6 and expansion of regulatory T cells in patients with cancer. These results suggest the possibility that the presence of periodontitis might contribute to cancer progression by inducing IL-6-dependent tumor cell proliferation and suppressing anti-cancer immunity by inducing regulatory T cells (70). However, cytokine-driven systemic inflammation can also affect periodontitis activity. For instance, the presence of rheumatoid arthritis has been shown to increase the risk for periodontitis occurrence and this effect correlated with increased levels of IL-1beta and TNF in the gingival crevicular fluid of patients suffering from both disorders (71).

In this review, we will summarize the role of pro- and anti-inflammatory cytokines in disease pathogenesis and discuss potential areas for therapeutic intervention in periodontitis.

## Cytokines in the pathogenesis of periodontitis

### Interleukin-1

Interleukin-1 (IL-1) is a pro-inflammatory cytokine with potent immunoregulatory functions in chronic periodontitis (72, 73). IL-1alpha and IL-1beta can by released during cell damage and activation of immune cells such as macrophages. These cytokines become fully activated by proteases (e.g. caspase1) in the extracellular space that are released by local immune cells and serve as alarmins to initiate recruitment and activation of IL-1R1-expressing immune cells. Hereby, they control innate immune responses, inflammasome activation and T cell driven immune responses.

Several lines of evidence suggest an important role of IL-1 in patients with periodontitis. First, IL-1 gene polymorphisms (e.g. coding variants for Lys3, Asn3 and Met 256) are correlated with the risk for development of periodontitis (74). Second, IL-1 levels in the gingival crevicular fluid were significantly higher in patients with chronic periodontitis as compared to periodontally healthy subjects (75). Particularly high levels were noted in elder patients as compared to younger patients (73, 76), although no statistically significant difference between systemic IL-1beta levels were observed between patients (40 years or younger) with chronic periodontitis and aggressive periodontitis (77). Finally, in patients with periodontitis, a significant decrease in levels of IL-1beta was observed after non-surgical periodontal treatment suggesting that successful therapy prevents disease progression at least in part by suppression of IL-1 function (75, 78).

In chronic periodontitis, IL-1 as well as the pro-inflammatory cytokine TNF have been suggested to control the spread of an inflammatory front to deeper areas in the connective tissue where they drive loss of connective tissue attachment, osteoclast activation and subsequent loss of alveolar bone (72). Therefore, IL-1 plays a predominant role in mediating periodontal tissue destruction. Accordingly, studies in the ligature-induced periodontal model indicated a key role of IL-1 in periodontitis. IL-1beta was rapidly induced in models of experimental periodontitis (heat-killed bacterial-induced periodontitis and ligature-induced periodontitis) and contributed to local and systemic inflammation in this disease (66). Moreover, functional studies showed that inactivation of the IL-1 inhibitor interleukin - 1 receptor antagonist (IL-1RA) drives disease activity. Specifically, Il1ra^-/-^ mice showed more bone loss than that of wild-type control mice associated with an expansion of IL-17 expressing immune cells and an enrichment of Il17, IL-17-associated transcripts (Il1b, Il6, Il23, Tgfb) and Rank. These findings suggested that IL-1RA plays a protective role in parodontitis by suppressing an IL-1-dependent hyper-IL-17 response in the gingiva that plays a crucial role in disease pathogenesis (79). However, a complete absence of IL-1 has also been suggested to cause pro-inflammatory effects in periodontitis, as this cytokine also limits bacterial dissemination (80).

### Interleukin-6

IL-6 is a key regulatory cytokine that affects chronic inflammatory processes by binding to target cells via the membrane bound IL-6R. However, cells lacking this receptor can also respond to IL-6 upon binding of IL-6 to the soluble IL-6R followed by activation of gp130 on target cells (IL-6 trans-signaling) (64, 65, 81). By using these mechanisms IL-6 can activate numerous types of target cells in chronic periodontitis.

In humans, IL-6 levels in gingival crevicular fluid levels were significantly higher in subjects diagnosed with chronic periodontitis as compared to periodontally healthy control subjects (75, 82). Studies in patients showed that IL-6 is expressed in apical periodontitis, where its levels correlate to the size of the periapical lesions (83). Particularly high IL-6 levels were observed in elder patients with periodontitis (73). However, no difference between systemic IL-6 levels in young patients (40 years or younger) were noted netween chronic periodontitis and aggressive periodontitis (77). In apical periodontitis, Porphyromonas gingivalis was detected in human lesional tissue in correlation with IL-6/STAT3 expression and activation of M1-like macrophages suggesting that bacteria may induce IL-6 production and immune cell activation via the transcription factor STAT3 (84). The presence of periodontitis and cancer has also been suggested to synergistically increase IL-6 levels in serum of affected patients (70). Additionally, it was found that the levels of salivary IL-6 are significantly induced in patients with calculus associated chronic periodontitis as compared to healthy control subjects and cytokine levels were correlated to disease progression (85). In addition, immunohistochemistry of lesions in advanced periodontitis showed the presence of CD4+ T cells co-expressing IL-6 (86). Some studies also reported a decrease of IL-6 in the gingival crevicular fluid after nonsurgical periodontal therapy suggesting that IL-6 may serve as a biomarker for response to therapy (78, 87, 88). Similarly, another study in patients with malocclusion secondary to periodontitis, used traditional straight-wire orthodontic treatment and invisible orthodontic treatment without brackets for therapy and found reduced levels of IL-6 in gingival crevicular fluid and serum after 6 months of therapy (89). Finally, a recent analysis looked at the effect of non-surgical periodontal therapy on IL-6 and reported decreased serum levels after three months in individuals with obesity (7).

IL-6 is mainly produced by locally activated immune cells in periodontitis, such as neutrophils and macrophages resident in periodontitis lesions which can produce IL-6 ex vivo after bacterial stimulation (83). In experimental models of periodontitis, IL-6 is highly expressed in lesions and several cell types including immune cells and osteoblasts contribute to its production. It has been suggested that IL-6 activates pro-inflammatory immune circuits in periodontitis by activating innate and adaptive immune cells and by favoring bone resorption together with IL-1 and TNF. Consistently, in experimental periodontitis, infection with Porphyromonas gingivalis induced IL-6 levels and consecutive activation of STAT3 and M1-like macrophages (84). Blockade of IL-6-dependent STAT3 activation *in vivo* inhibited periapical bone resorption and apical infiltration of immune cells such as macrophages highlighting the potential therapeutic relevance of targeting the IL-6/STAT3 signaling pathway (84). Consistently, treatment of experimental ligature-induced periodontitis by systemic administration of the humanized monoclonal IL-6R antibody tocilizumab suppressed inflammatory cell recruitment, Th17 cytokines and impaired RANKL expression. Moreover, such treatment reduced alveolar bone resorption and attachment loss suggesting that IL-6R blockade may be a promising approach for periodontitis (90). However, in spite of these pro-inflammatory functions of IL-6, studies using IL-6 knockout mice reported more pronounced periapical lesions as compared to wild-type control mice suggesting that IL-6 can also exert anti-inflammatory functions possibly by controlling epithelial homeostasis and barrier integrity (83).

## Tumor necrosis factor

TNF is a pro-inflammatory cytokine mainly produced by immune cells such as macrophages and lymphocytes that occurs in soluble and membrane-bound forms (81). It acts by binding to two distinct types of TNF receptors, denoted TNFR1 and TNFR2, and thereby modulates various key pathways in inflammation including recruitment, activation and survival of immune cells, structural damage and angiogenesis (91).

Numerous studies have highlighted an important role of TNF in the pathogenesis of chronic periodontitis. Augmented levels of TNF protein were detected in chronic apical periodontitis lesions by immunohistochemistry, particularly in older patients, suggesting local induction of TNF levels during periodontitis (73). Moreover, increased serum levels of soluble TNF were observed in patients with periodontitis as compared to healthy controls and were associated with dental plaque load (Aggregatibacter actinomycetemcomitans and Porphyromonas gingivalis) (92). Inflammation-induced upregulation of systemic TNF levels (along with IL-1beta levels) has also been suggested to augment the risk for periodontitis in patients suffering from rheumatoid arthritis (71). However, non-surgical therapy of periodontitis did not result in suppression of TNF levels in gingival crevicular fluid in one study (78), while another study reported that treatment of malocclusion secondary to periodontitis reduced the levels of TNF in gingival crevicular fluid and serum at 6 months (89). Clinical studies on the use of TNF blockers such as infliximab (chimeric anti-human TNF antibody) suggested the clinical relevance of TNF in controlling periodontal parameters (93). In patients with rheumatoid arthritis receiving TNF blockers, lower periodontal indices including plaque index, gingival index, probing depth, clinical attachment loss, and bleeding on probing were found. Furthermore, treatment was associated with reduced TNF levels in gingival crevicular fluids. Thus, suppression of proinflammatory cytokines might prove beneficial in suppressing periodontal diseases (93). Another study in patients with rheumatoid arthritis receiving the fully humanized anti-TNF monoclonal antibody adalimumab indicated beneficial effects of therapy on periodontal parameters. Specifically, three months after therapy, anti-TNF-treated patients showed a marked decrease in gingival index, bleeding on probing and probing depth. This effect was associated with reduced serum levels of TNF and IL-6 (94).

In experimental periodontitis, increased local and systemic TNF levels were reported (66). The role of TNF signaling was further studied in a murine model of apical periodontitis induced by root canal infection by using tumor necrosis factor-α receptor-1 (TNFR1) knockout mice. These mice showed impaired activity of periodontitis with reduced recruitment of neutrophils, lower RANKL expression and lower numbers of activated osteoclasts as compared to wild-type mice indicating an important pro-inflammatory role of the TNF-TNFR1 signaling pathway in this disease (95). Consistently, another report showed that TNF blockade reduces periodontitis activity. In this report, etanercept, a human dimeric fusion protein blocking TNF, was tested in ligature-induced experimental periodontitis in rats (96). Etanercept treatment reduced neutrophil recruitment, ameliorated the degree of inflammation in periodontitis and reduced local tissue injury suggesting that TNF drives local inflammation and tissue injury in periodontitis. Based on these findings, TNF appears to regulate the local spread of inflammation to deeper areas thereby favoring loss of connective tissue attachment and loss of alveolar bone (72).

## IFN-gamma

IFN-gamma is a potent regulatory cytokine that is produced by immune cells such as lymphocytes. It activates target cells via binding to a specific receptor containing IFN-gammaR1 and IFN-gammaR2 proteins which is expressed on many cells including macrophages and epithelial cells (97, 98). In the context of periodontitis, IFN-gamma has been suggested to mediate several pro-inflammatory functions by amplifying local immune responses and favoring bone remodeling.

Several studies indicated an increase of serum levels of IFN-gamma in periodontitis. One study reported increased serum levels of IFN-gamma, TNF, and IL-10 in patients with periodontitis as compared to healthy controls (92). Interestingly, increased serum levels of IFN-gamma were associated with increased local dental plaque load suggesting that bacterial antigens induce IFN-gamma levels (92). Moreover, some studies indicated increased levels of this cytokine in gingival crevicular fluid in periodontitis, whereas other studies did not find significant differences in this disease to healthy controls (75, 99). However, immunohistochemistry of lesions in advanced periodontitis showed the presence of CD4+ T cells co-expressing IFN-gamma suggesting the presence of Th1 T cells (86). In additon, higher levels of IFN-gamma in interdental gingival specimens by immunohistochemistry were noted in patients with IFNgamma +874A/T gene polymorphisms as compared to controls and the former patients have a higher risk of chronic periodontitis (100) suggesting a pathogenic role of this cytokine in the pathogenesis and progression of the disease. Specifically, IFN-gamma has been suggested to control osteoclastogenesis thereby favoring bone resorption and to induce production of neopterin by macrophages which protects them against oxidative stress and cell death at the site of periodontal inflammation (99, 101).

In experimental periodontitis, higher levels of IFN-gammaR1 were noted at an early stage of active disease rather than during periodontal disease progression (102). Moreover, an induction of IFN-gamma was observed in this disease. Functionally, IFN-gamma deficient mice showed reduced periodontitis in response to Aggregatibacter actinomycetemcomitans or Porphyromonas gingivalis infection (103, 104). This finding was associated with lower alveolar bone loss highlighting the role of IFN-gamma in osteoclastogenesis and reduced local levels of pro-inflammatory cytokines such as IL-1 and TNF. However, an increased bacterial load in periodontal tissues and a disseminated bacterial infection was noted in IFN-gamma knockout mice underling the role of this cytokine in controlling bacterial dissemination (103).

## Interleukin-10

IL-10 is a crucial cytokine with anti-inflammatory functions that is produced by various cell types, including lymphocytes, macrophages and epithelial cells (105). It limits the host immune response to pathogens and prevents the overactivation of inflammatory processes by binding to IL-10R-expressing target cells, thereby preventing tissue damage in the host (106). In this context, IL-10 inhibits effector T cell and macrophage activation leading to reduced production of pro-inflammatory cytokines such as IFN-gamma, IL-1, TNF, and IL-6. Furthermore, IL-10 inhibits osteoclastic bone resorption and regulates osteoblastic bone formation thereby affecting bone function (107). The receptor for IL-10 is a complex comprising two IL10Rα (IL10R1) molecules (encoded by the *Il10ra* gene) and two IL10Rβ (IL10R2) molecules (encoded by the *Il10rb* gene) (108). Low expression of IL-10 is associated with the development of immunopathology in response to infection and an increased risk for the development of chronic inflammatory diseases such as periodontitis (106).

In patients with periodontitis, IL-10 gene polymorphisms have been identified, underlining the potential relevance of IL-10 in this disease. For instance, the IL10–1082 polymorphism and the IL-10 -592C>A polymorphism were identified as a putative risk factors for chronic periodontitis (109, 110). Moreover, variants in the IL-10 promoter gene have been associated with a predisposition to chronic periodontitis (111). Some studies reported increased serum levels of IL-10 in chronic periodontitis. However, IL-10 serum levels were similar between chronic periodontitis and aggressive periodontitis (77). Another study reported a trend toward higher IL-10 levels in older patients as compared to younger subjects with periodontitis (76). In addition, higher levels of IL-10 were observed in the gingival crevicular fluid and in the inflamed tissue of periodontitis patients along with the presence of putative IL-10-producing cells, such as CD4+CD25+FoxP3+ regulatory T cells, in the inflammatory infiltrate of gingival tissues (112).

The important role of IL-10 was underscored by studies in genetically engineered mice showing that IL-10-deficient mice exhibit a hyperinflammatory phenotype and are highly susceptible to Porphyromonas gingivalis-induced periodontitis (113). This effect was mediated by an excessive IL-12p40 driven myeloid immune response in the absence of IL-10. In an experimental periodontitis model, adoptively transferred IL-10-producing B cells demonstrated the ability to reduce local IL-17 and RANKL expression and alleviate alveolar bone resorption by reducing periodontal osteoclastogenesis. These findings suggest that IL-10 may serve as a potential therapeutic agent for the prevention of inflammatory damage to alveolar bone in periodontitis, through the reduction of pro-inflammatory cytokine expression and the inhibition of local Th17 cell proliferation (114). Similarly, another study reported that IL-10 dampens an excessive IL-17-mediated pro-inflammatory cytokine circuit in Porphyromonas gingivalis–induced experimental murine periodontitis and ligature-induced alveolar bone-loss models (115). In addition, it was demonstrated that exosomes derived from reparative M2-like macrophages contain IL-10 mRNA and possess the capacity to promote osteogenesis and to suppress osteoclastogenesis in alveolar bone in an IL-10-dependent manner. This suggests that IL-10 is a potent modulator of periodontitis-induced bone damage (116).

## Interleukin-23

IL-23 is a heterodimeric cytokine of the IL-12 family that consists of two subunits, designated p19 and p40 (117, 118). The p40 subunit is shared with the heterodimeric cytokine IL-12 (p35/p40) (119–121). IL-23 is produced by various cells, including antigen-presenting cells such as macrophages, in response to bacterial or viral infections. In terms of its functional properties, IL-23 can stimulate the production of pro-inflammatory cytokines by innate lymphoid cells and Th17 cells, thereby initiating several pro-inflammatory circuits in the context of inflammatory disorders such as periodontitis (122, 123).

Several studied assessed the production of IL-23 in patients with chronic periodontitis (123). The majority of studies have demonstrated an elevation in the levels of IL-23 and IL-17 in the gingival crevicular fluid of individuals with periodontitis and gingivitis in comparison to healthy controls (20, 124–127). Furthermore, the levels of the IL-23/IL-17 axis have been found to be positively correlated with the progression and severity of periodontal disease, as determined by probing depth, clinical attachment level, and gingival index. Furthermore, a positive correlation was observed between the levels of both IL-23 and IL-17, which is consistent with the concept that IL-23 can induce the expression of IL-17 and clonal expansion of Th17 cells during disease progression (20). Importantly, a recent study demonstrated IL-23 production by non-immune cells in periodontitis (128). In patients with chronic periodontitis and periodontitis associated with genetic immune deficiencies, such as leukocyte adhesion deficiency type 1 (LAD1), IL-23 was highly expressed by epithelial cells in close proximity to periodontal tissues suggesting that epithelial-derived IL-23 controls disease activity (**Figure 1**) (129). In human periodontal ligament fibroblasts, the Th17-polarizing cytokine IL-1β was identified as an important regulator of IL-23 19 expression by upregulating the expression of p19 through NF-κB signaling and MAPKs-dependent AP-1 pathways (130). Finally, another study reported increased salivary concentrations of IL-17A and IL-23 in patients with localized periodontitis as compared to controls and patients with generalized periodontitis (131).

In experimental models of periodontitis, an epithelial cell-intrinsic production of IL-23 was noted that was critically dependent on the local, disease-associated microbiome and TLR5 signaling (129). Epithelial IL-23 expression localized to areas proximal to the disease-associated microbiome. Functional studies using bone marrow chimeric mice from Il23a+/+ and Il23a-/- mice and adoptive transfer studies between these mouse strains demonstrated that non-hematopoietic-cell-derived IL-23 mediates inflammation and disease pathology in experimental periodontitis (129). Another study found that IL-23 p19 was produced by CD68+ macrophages in experimental periodontitis, and *in vitro* generated CD68+ cells could be stimulated by Porphyromonas gingivalis-derived lipopolysaccharide to produce p19 mRNA suggesting that TLR signaling controls IL-23 production (132). Functional studies indicated that IL-23 can stimulate RANKL production in osteoblasts and drive osteoclast development in inflammation-mediated bone pathology, thereby favoring bone loss in periodontitis (133). Furthermore, p40-deficient mice lacking IL-12 and IL-23 exhibited more advanced tissue destruction and a reduced inflammatory cell infiltrate after Porphyromonas gingivalis challenge indicating an important role for these cytokines in controlling disease activity (47).

## Interleukin-17 and Th17 cells

The IL-17 family comprises six pro-inflammatory cystine knot cytokines, designated IL-17A-F (134). Among these cytokines IL-17A and IL-17F have been proposed to play a pivotal role in inflammatory disorders such as chronic periodontitis. Both cytokines are produced by lymphocytes and innate immune cells such as innate lymphoid cells in response to stimulation with IL-1beta or IL-23 (135). They function to regulate protective immune responses against fungal infections and to drive neutrophil recruitment and enhanced barrier function at mucosal surfaces by binding to IL-17R-expressing target cells (134). The IL-17R family comprises five subunits, designated IL-17RA, IL-17RB, IL-17RC, IL-17RD, and IL-17RE.

In patients with periodontitis, flow cytometry analyses of blood samples revealed an expansion of IL-17A+IL-17F- and IL-17A-IL-17F+ Th17 cells as compared to healthy controls (136). Moreover, further analysis demonstrated a high correlation between IL-17A+IL-17F- Th17 cells and attachment loss or probing depth suggesting that these IL-17 producing cells are involved in the pathogenesis of periodontitis (136). Additional studies assessed the expression of IL-17 in salivary samples. A recent study found that patients with stage I and II periodontitis exhibited elevated levels of salivary IL-17 compared to healthy controls. In contrast, a significant decrease in stage III was observed compared to the control patients (137). Nevertheless, a separate study indicated that the levels of salivary IL-17 were markedly elevated in patients with calculus-associated chronic periodontitis in comparison to healthy controls. Furthermore, these levels exhibited a discernible increase with the advancement of the disease (85). In addition to these cells, other studies looked at the expression of IL-17 in gingival crevicular fluid and inflamed lesion in periodontitis patients and most studies reported elevated IL-17 levels (138–140). In the inflamed tissue, a strong infiltration of IL-17-producing T cells was observed in the bottom region of chronic periodontitis lesions, while the Th17-inducing cytokine IL-23 was locally produced by CD68+ macrophages (132). Expression of both cytokines was mainly detected in periodontal lesions, especially in the tissue adjacent to bone destruction in periodontitis (141).

The functional role of IL-17 was additionally studied in murine models of periodontitis. In experimental ligation-induced periodontitis, the pro-inflammatory role of IL-17 has been described. IL-10 and IL-1RA have been identified as important soluble mediators controlling IL-17 levels. Mice deficient in IL-10 exhibited greater bone loss, which was accompanied by elevated levels of IL-17 and IL-17-mediated chemokine and cytokine expression in both the Porphyromonas gingivalis-induced experimental murine periodontitis model and the ligature-induced alveolar bone loss model (115). Furthermore, an increased expression of IL-17 has been noted that is controlled by IL-1RA function. Specifically, Il1ra^-/-^ mice showed an expansion of IL-17 producing T cells with marked alveolar bone loss. Anti-IL-17 antibody treatment reduced the alveolar bone loss indicating that IL-17 plays a pivotal role in regulating bone metabolism (79). Therefore, IL-17, along with IL-1β, IL-6, and TNF, belongs to a group of pro-inflammatory cytokines in this disease that can trigger osteoclast activation, resulting in bone resorption. IL-17 has also been identified as a key player in the induction of neutrophil-mediated inflammation and bone loss in periodontitis. Neutrophil extracellular traps derived from neutrophils have been demonstrated to be early triggers of pathogenic mucosal inflammation and bone destruction in experimental periodontitis, through the upregulation of IL-17/Th17 responses (142). Despite these findings, other reports have indicated that IL-17 signaling and the IL-17R adapter tumor necrosis factor receptor-associated factor 3 interacting protein 2 (TRAF3IP2) play an essential role in preventing pathogen-induced bone destruction in experimental periodontitis. This is evidenced by the observation that IL-17RA- and TRAF3IP2-deficient mice exhibited enhanced periodontal bone loss upon infection with Porphyromonas gingivalis, which may be attributed to impaired neutrophil function and altered host defense (143–145).

It has been postulated that periodontitis-derived IL-17 may contribute to the exacerbation of other chronic inflammatory conditions. This hypothesis is supported by findings in mice with experimentally-induced arthritis that were exposed to Porphyromonas gingivalis, which exhibited heightened joint damage and Th17 cell frequencies when compared to non-infected mice. This was accompanied by elevated TNF and IL-17 production and articular neutrophil infiltration. In contrast, arthritis aggravation and changes in neutrophil infiltration were absent in IL-17RA-deficient mice (67).

The above data collectively highlight the pivotal role of IL-23 and IL-17 in the pathogenesis of periodontitis, with particular emphasis on the crucial involvement of IL-23R-expressing Th17 cells (20, 146). It is important to note, however, that Th17 cells do not exclusively play a proinflammatory role in periodontitis due to the presence of marked T cell plasticity. In experimental periodontitis, immunoregulatory Th17 cells have been identified through IL-17 fate mapping experiments (147). In contrast to the local proinflammatory Th17 cells in periodontitis, these cells reside in the gingiva draining lymph nodes where they acquire features of T follicular helper (Tfh) cells. Blocking the plasticity of Th17 cells into Tfh cells resulted in increased periodontal bone loss, reduced IgG levels in the oral cavity, and impaired capacity to restrict the biomass of the oral commensal community (147). Despite these findings, local Th17 cells play a crucial pro-inflammatory role in periodontitis, and the cytokines produced by these cells, such as IL-17, represent an intriguing potential therapeutic target.

## Mechanisms of action: cytokines as a link between periodontitis and gut inflammation

Recent studies have suggested a potential link between periodontitis and gut inflammation, which is controlled by the microbiome and cytokines induced by microbial factors. Specifically, studies have demonstrated that oral bacteria, particularly periodontopathic bacteria, play a role in inducing dysbiosis of the gut microbiota, which in turn leads to the development of gut dysbiosis-related pathology (148). Conversely, the disruption of the gut microbiota has been found to have a negative impact on the pathogenesis of periodontal disease (148). In experimental ligature-induced periodontitis in Wistar rats, an overexpression of Th1/Th2-related cytokines was associated with a moderate infiltrate of inflammatory cells in the colon. In particular, higher expression levels of IFN-gamma, IL-1alpha/beta, IL-2, IL-4, IL-5, IL-10, IL-12, IL-13, and TNF were observed in the intestinal tissues. The results indicated that periodontitis was associated with increased intestinal inflammation compared to controls. This suggests that periodontitis may exacerbate intestinal inflammation, potentially by modulating the intestinal microbiota, which in turn induces cytokine production and local inflammation (149). Another study demonstrated that periodontitis results in the expansion of oral pathobionts, including Klebsiella and Enterobacter species, within the oral cavity. These organisms are subsequently ingested and transported to the gut, where they activate the inflammasome in colonic mononuclear phagocytes, triggering inflammation (150). However, periodontitis affects gut inflammation through a second mechanism, the generation of oral pathobiont-reactive Th17 cells. These cells can home to the inflamed gut through alpha4/beta7 integrins on their surface and interaction with addressins on gut endothelial cells. Oral pathobiont-reactive Th17 cells are imprinted with gut tropism and thus migrate to the inflamed gut. Consequently, periodontitis can exacerbate intestinal inflammation through the translocation of colitogenic pathobionts and the trafficking of pathogenic T cells (150). However, it is also the case that gut-derived Th17 cells can affect the activity of periodontitis. In particular, the translocation of the oral pathobiont Porphyromonas gingivalis from the gut to the mouth can exacerbate oral pathobiont-induced periodontitis through the action of Th17 cells (151). These Th17 cells are differentiated in Peyer’s patches and can then migrate to and accumulate in the mouth upon oral infection. These findings indicate that the activity of periodontitis induced by oral pathobiont-responsive Th17 cells is regulated by the intestinal microbiome. Consequently, alterations in the intestinal microbiome composition may influence the development of periodontitis (151).

## Clinical data and perspectives

**Figure 2:**
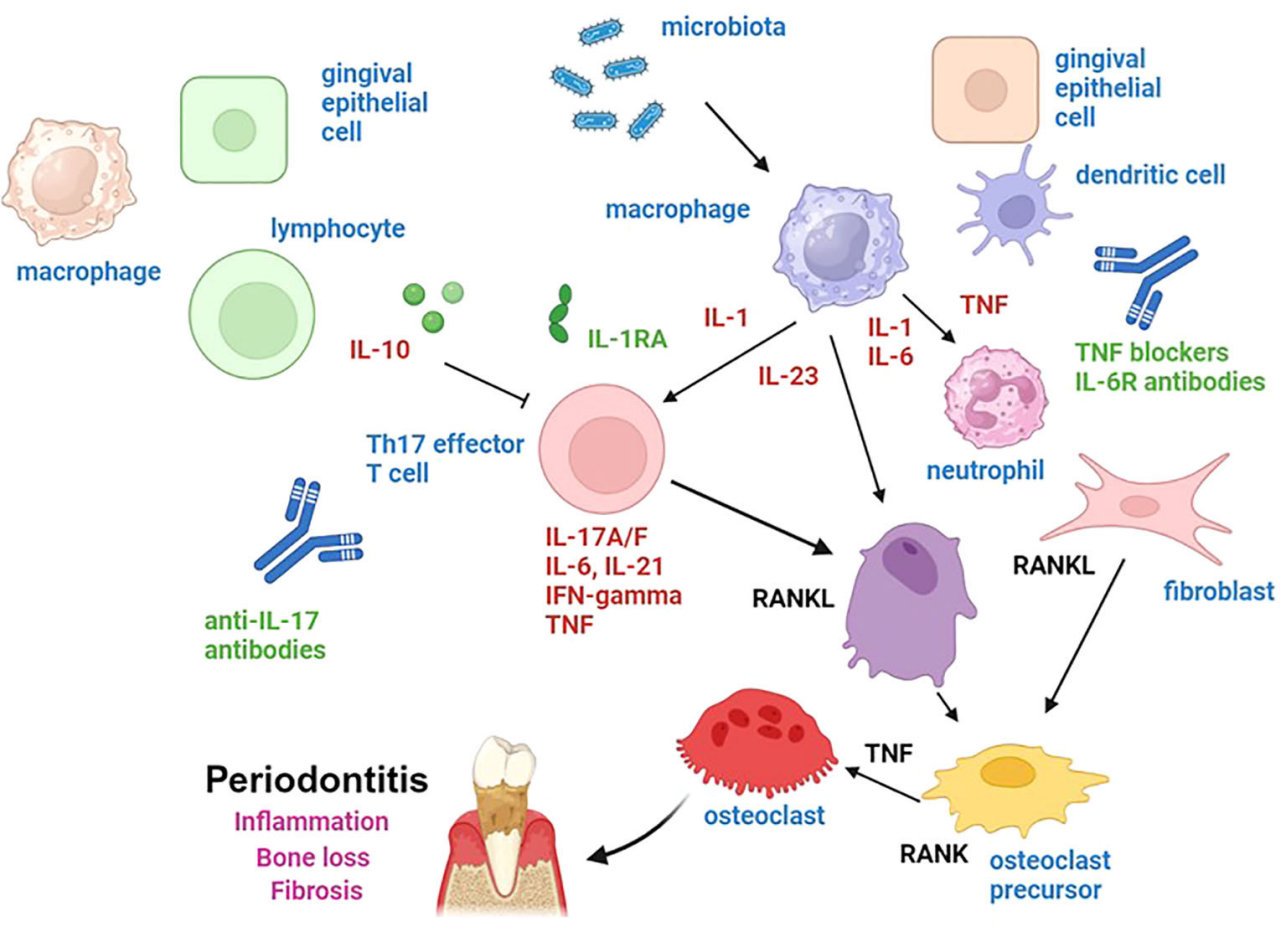
Potential therapeutic avenues for periodontitis based on modulation of cytokine function have been identified. In experimental models of periodontitis, several strategies modulating cytokine function have been shown to suppress inflammation and bone loss. Administration of IL-17 antibodies led to suppression of inflammatory activity in experimental periodontitis. Moreover, IL-1RA and IL-6R blockade (via tocilizumab) have been shown to block Th17 cell activity and local inflammation. Furthermore, IL-10 has been identified as an important suppressor of Th17 activity, suggesting that strategies for inducing IL-10 expression or function might be used for periodontitis therapy. Finally, studies in experimental periodontitis and patients with periodontitis have suggested that administration of TNF blockers such as etanercept, adalimumab, and infliximab might be used to suppress inflammation in periodontitis. Figure was created with BioRender.

The pivotal role of cytokines in the pathogenesis of periodontitis is substantiated by a multitude of studies that have employed patient samples and experimental models of periodontitis (6, 7, 46, 76, 87, 149, 152). These studies demonstrated that the balance between pro- and anti-inflammatory cytokines is altered in patients with periodontitis, thereby favoring inflammatory processes. Additionally, these studies revealed that cytokines play a pivotal role in local tissue destruction, bone loss, and systemic inflammation in this disease. Furthermore, it was demonstrated that modulation of cytokine expression or function can be utilized for therapy in experimental periodontitis. These findings suggest new avenues for future therapy of patients with periodontitis by regulating cytokine expression or function (**Figure 2**). The concept that modulation of cytokine function can be useful for therapy is supported by a number of lines of evidence. Firstly, studies in experimental models of periodontitis showed that suppression of pro-inflammatory cytokines such as IL-17A or activation of anti-inflammatory cytokines such as IL-10 can ameliorate inflammation and bone loss (79, 114–116). In addition, local therapy of patients with periodontitis has been shown to reduce local and systemic cytokine levels. For instance, in one study in patients with stage III periodontitis, non-surgical periodontal therapy caused a significant reduction in the levels of serum pro-inflammatory cytokines IL-6 and IL-8 (87). Furthermore, the administration of calcium hydroxide intracanal medications in conjunction with chlorhexidine gel has been demonstrated to markedly reduce cytokine levels, including IL-12, IL-17, IL-21, and IFN-gamma, while concurrently increasing IL-4, IL-5, IL-10, and IL-13 levels (153). Moreover, although direct prospective trials on the use of anti-cytokine agents and biologicals targeting cytokine function have not yet been conducted, studies in patients with concomitant disorders have indicated the potential of anti-cytokine drugs to modulate disease activity in periodontitis. Specifically, treatment of patients with rheumatoid arthritis and periodontitis with TNF blockers such as adalimumab or infliximab has been shown to improve periodontal indices and reduce TNF levels in gingival crevicular fluids (93, 94). These findings provide a rationale for further investigation into the role of cytokines in patients with periodontitis *in vivo*. Such studies may inform the development of more effective therapeutic strategies for this disease.
